# Comparing the prognosis of esophageal adenocarcinoma with bone and liver metastases: A competing risk analysis

**DOI:** 10.1371/journal.pone.0303842

**Published:** 2024-09-25

**Authors:** Xinglian Zhu, Mingxing Mo, Shaojun Zheng, Kunning Han, Guoyang Li, Fang Zhao

**Affiliations:** 1 Department of Respiratory, Panyu Hexian Memorial Hospital of Guangzhou, China; 2 The Third Affiliated Hospital of Guangzhou Medical University, Guangzhou, China; 3 Guangdong Provincial Key Laboratory of Infectious Diseases and Molecular Immunopathology, Institute of Oncologic Pathology, Cancer Research Center, Shantou University Medical College, Shantou, China; 4 Department of Neurology, Shenzhen People’s Hospital, Shenzhen, China; 5 Food Inspection and Quarantine Technology Center of Shenzhen Customs District, Shenzhen, China; University of Michigan, EGYPT

## Abstract

**Background:**

About half of the patients with esophageal cancer are presenting with metastasis at initial diagnosis. However, few studies have concerned on the prognostic factors of metastatic esophageal adenocarcinoma (mEAC). This research aimed to investigate the effects of single bone metastasis (BM) and single liver metastasis (LM) on prognosis of mEAC patients.

**Methods:**

Data were obtained from the National Cancer Institute’s Surveillance, Epidemiology, and End Results (SEER) Program database. We compared the effects of LM and BM on overall survival (OS), EAC-specific survival (CSS), and EAC-specific death (EASD) by multivariate Cox regression, Kaplan-Meier analysis, and competing risk regression models.

**Results:**

A total of 1,278 EAC patients were recruited in this study. Of which 78.95% (1009/1278) were EASD, and 12.68% (162/1278) were non-EAC-specific death (non-EASD). In multivariate Cox regression analysis, surgery, chemotherapy, and AJCC.T2 (*vs*. T1) were identified as protective factors for OS&CSS, while divorced/separated, single/unmarried (*vs*. married), grade III-IV (*vs*. grade I-II) and BM (*vs*. LM) were identified as risk factors. Competing risk regression analysis further confirmed that surgery and chemotherapy were beneficial to the patients with mEAC, and BM (*vs*. LM) was a risk factor for mEAC patients when considering the existence of the competitive risk events.

**Conclusion:**

Our study indicated that mEAC patients with BM face a worse prognosis compared to those with LM. Additionally, surgery and chemotherapy emerge as protective factors for mEAC patients. These findings offer evidence-based insights for clinical management and contribute to the field.

## Introduction

Esophageal cancer (EC) is among the top 10 leading causes of cancer-related death worldwide [1]. EC accounts for about 4% of all cancers in the United States [2]. It is estimated that 13,460 new cases and 12,720 deaths are caused by EC every year [3]. Esophageal squamous cell carcinoma (ESCC) and esophageal adenocarcinoma (EAC) are two main histological subtypes of EC. While ESCC has been the subject of abundant research [4, 5], EAC, the other subtype of esophageal cancers, has not received much attention and investigation in the past time. However, EAC now comprises up to 80% of EC in the United States [6]. EAC has a 5-year survival rate of less than 20% and most patients have locally advanced or widespread metastatic disease at first diagnosis. Consequently, most EAC patients are suffering treatment resistance [7]. Even when tumors are superficial, EAC patients have a high risk of metastasis, which leads to poor outcomes [8]. Nearly 50% of EC patients are presented with distant metastases at first diagnosis [9]. The prognosis of EAC patients with metastasis is poor, with a median survival time of less than 10 months. According to the previous studies, the median survival time of EAC patients with liver, lung, and bone metastasis are 5 months, 6 months, and 4 months, respectively [7, 10, 11]. Therefore, the difference in prognosis varies significantly depending on the site of metastasis. Age, race, type of pathology, metastatic pattern, treatment strategy and other factors might have impact on the prognosis of patients with metastatic EAC [12, 13].

Several studies have explored the impact of distant metastasis on the prognosis of EAC, using by traditional methods like Kaplan-Meier and Cox proportional approach. For example, Qiu et al. found that EAC patients with multiple organ metastasis had worse overall survival and cancer-specific survival than those with single organ metastasis [14]. Compared with different metastatic patterns, Shou et al. discovered that liver metastasis had the worst prognosis, while lung metastasis had the best prognosis among EAC patients [15]. However, these studies neglecting the impact of competing risk events on the prognosis of EAC. A competing risk is an event whose occurrence precludes the critical event of interest. Competing event analysis is time-to-event analysis that takes into account all kinds of fatal or non-fatal events that might change or stop subjects from reaching the interest endpoint [16]. This method can provide clinicians with a more accurate and less biased prediction of the outcome of disease, and help them to make individualized therapy strategies [17].

In this study, a competing-risk analysis was conducted to investigate the distant metastasis (DM) patterns and prognosis of EAC patients with LM and BM in Surveillance Epidemiology and End Results (SEER) database, using cumulative incidence function (CIF) to show each probability of each event, and Gray’ s test to discriminate the impact of risk factors on specific events for the first time, systematically compared the impact of BM and LM on the prognosis of EAC.

## Methods

### Data source

All patients of EAC with single liver and bone metastasis were diagnosed between 2010 and 2015 using anonymized data from the SEER database: Incidence·SEER 18 Regs Custom Data (with additional treatment fields. Nov 2018 Sub (1975–2016 varying) by SEER‐Stat software (SEER*Stat 8.3.8). SEER currently collects and publishes data on cancer incidence and survival rates, population-based cancer registries that cover nearly 30% of population in the U.S. As the data used was from SEER dataset (public), Ethics approval and consent to participate could be checked in SEER.

### Study population

**Fig 1:**
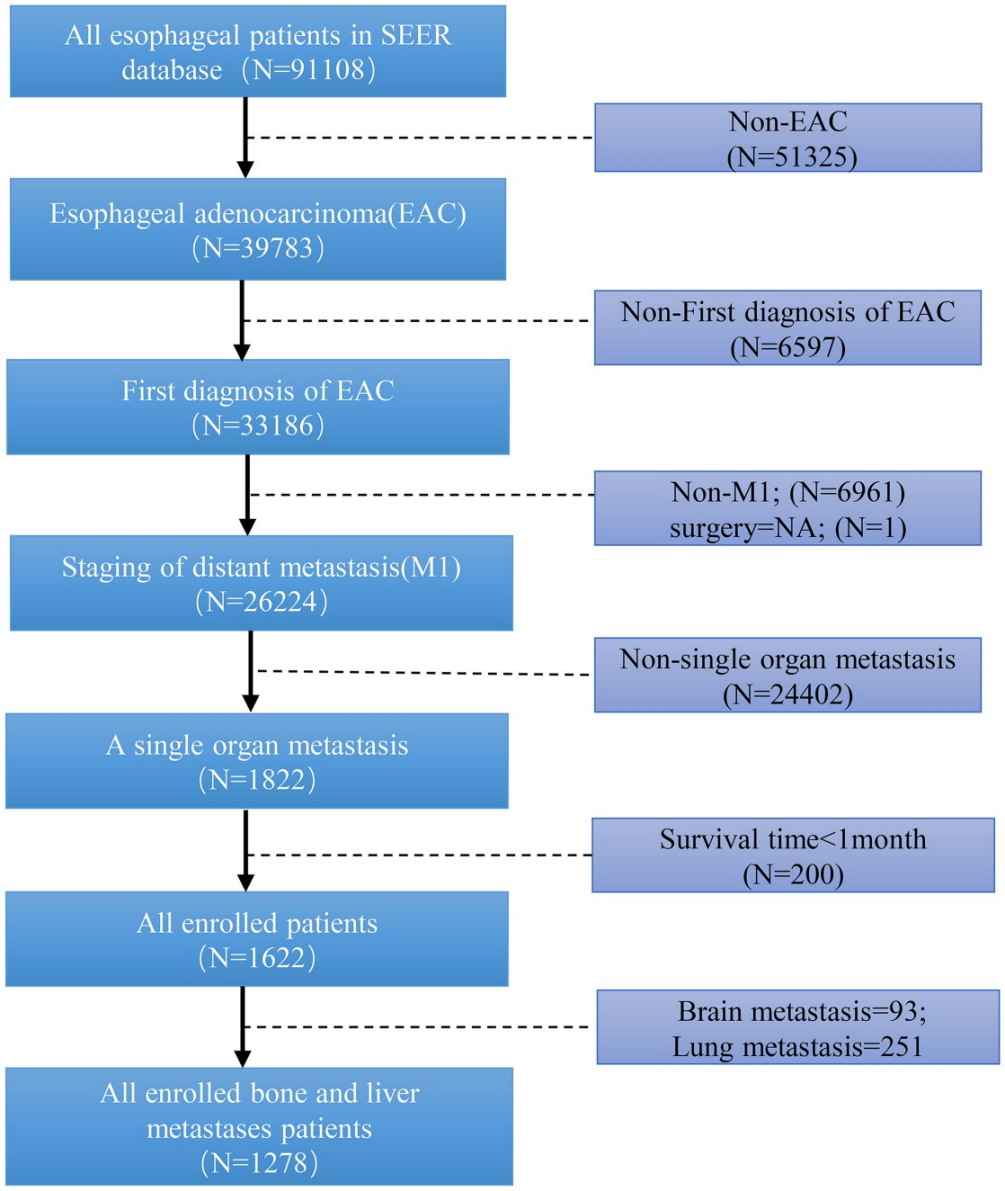
Flowchart of EAC patients with single-organ metastasis selection for this study.

Patients were eligible if they matched the following criteria: (1) with unequivocal American Joint Committee on Cancer (AJCC) stage; (2) with distant metastasis for liver or bone; (3) with follow-ups longer than 1 month; (4) with pathological types limited to EAC (8140/3) (Fig 1).

We collected the following information from each patient: age, sex, race, age at diagnosis, marital status, grade, AJCC stage, pathological tumor-node-metastasis (pTNM), radiation status, surgery status, chemotherapy status, survival time, primary site, and cause of death. The sixth edition of the AJCC staging system was adopted since it was employed for recording data in the SEER database from 2005. Finally, EAC patients with single bone and liver metastasis were included in our study.

### Statistical analysis

The primary end points were the single organ metastasis, CSS and OS. The main explanatory variable was survival with metastasis in different single organs (bone and liver). All the variables in this study were presented as categorical data, and the Chi-square test was applied to test differences in demographic distribution or clinical characteristics between two groups. Kaplan-Meier curve analysis was employed to evaluate the 1 year and 3 years CSS and OS rates, and the differences between single bone and liver metastasis were determined by log-rank test. We applied multivariate Cox regression model to explore the independent prognostic factors in OS and CSS.

Other causes of death in this study were taken as competing events in our analysis of competing risks. A competitive risk model was employed to identify factors affecting the cumulative incidence of EAC. Cumulative incidence function (CIF) was employed to show the probability of each event and Gray’s test was used to test the differences in the CIF between the two groups due to any competing risk events. In addition, we conducted the competing risk regression with non-cancer deaths as the competing risk and reweights the main outcome risk according to competing outcomes, with results presented by subdistribution HR (SHRs) and 95% confidence intervals (CIs).

In addition, we applied conditional survival (CS) to estimate the probability of survival for additional y years considering the actual survival time of x years. It can be calculated by this formula: CS (y | x) = S (x + y) / S (x) [18].

All statistical analyses were performed using R 4.0.5 (Lucent Technologies, USA), with the “cmprsk” package for competing risk analysis, the “survival” and “survminer” package for Kaplan‐Meier analysis. Two-tailed *P* < 0.05 was regarded statistically significant.

## Results

### Characteristics of the patients and sites of distant metastases

Among 1278 EAC patients with de novo stage IV, 952 (74.49%) EAC patients had liver metastasis and 326 (25.51%) had bone metastasis. All patients were diagnosed from 2010 to 2015, 324 (25.35%) were aged 23–55, 441 (34.51%) were aged 56–65, 193 (15.10%) were aged 66–70, 320 (25.04%) were aged 71–97. Most patients were males (87.87%) and whites (93.90%), and 751 (58.76%) got married, 1000 (78.25%) with tumor size 33–40 cm. 283 (22.14%) patients were in T1 status and 631 (49.37%) patients were in N1 status. Moreover, nearly half of the patients had grade III-IV (49.45%). Only 31 (2.43%) and 41 (3.21%) underwent surgery and radiotherapy, respectively. However, majority patients received chemotherapy (72.61%). 107 (8.37%) were censored, 1009 (78.95%) died of EAC and 162 (12.68%) died from competing-risk events, such as non-cancer diseases or traffic accidents. Compared with single-bone metastasis group, significant differences (*p* <0.05) were found in age at diagnosis, sex, race, marital status and primary tumor size. These results were shown in Table 1.

**Table 1:** Clinical features of EAC patients between liver and bone metastasis groups.

Variables	Total (n = 1278)	Liver metastasis (n = 952)	Bone metastasis (n = 326)	*p* *
**Age.cat, n (%)**				0.009
23~55	324 (25.35)	259 (27.21)	65 (19.94)	
56~65	441 (34.51)	314 (32.98)	127 (38.96)	
66~70	193 (15.1)	133 (13.97)	60 (18.4)	
71~97	320 (25.04)	246 (25.84)	74 (22.7)	
**Sex, n (%)**				0.114
Female	155 (12.13)	124 (13.03)	31 (9.51)	
Male	1123 (87.87)	828 (86.97)	295 (90.49)	
**Race, n (%)**				0.049
Black	50 (3.91)	40 (4.2)	10 (3.07)	
Others	28 (2.19)	26 (2.73)	2 (0.61)	
White	1200 (93.9)	886 (93.07)	314 (96.32)	
**Marital, n (%)**				0.358
Divorced/separated	151 (11.82)	115 (12.08)	36 (11.04)	
Married	751 (58.76)	546 (57.35)	205 (62.88)	
Single/UnMarried	225 (17.61)	173 (18.17)	52 (15.95)	
Widowed/Others	151 (11.82)	118 (12.39)	33 (10.12)	
**Diagnosis, n (%)**				0.517
2010~2012	629 (49.22)	463 (48.63)	166 (50.92)	
2013~2015	649 (50.78)	489 (51.37)	160 (49.08)	
**Primary. size, n (%)**				< 0.001
15~24cm	39 (3.05)	25 (2.63)	14 (4.29)	
25~32cm	60 (4.69)	30 (3.15)	30 (9.2)	
33~40cm	1000 (78.25)	771 (80.99)	229 (70.25)	
Others	179 (14.01)	126 (13.24)	53 (16.26)	
**Surgery, n (%)**				0.865
NO	1247 (97.57)	928 (97.48)	319 (97.85)	
YES	31 (2.43)	24 (2.52)	7 (2.15)	
**Radiotherapy, n (%)**				< 0.001
NO	1237 (96.79)	935 (98.21)	302 (92.64)	
YES	41 (3.21)	17 (1.79)	24 (7.36)	
**Chemotherapy, n (%)**				0.009
NO	350 (27.39)	242 (25.42)	108 (33.13)	
YES	928 (72.61)	710 (74.58)	218 (66.87)	
**AJCC.T, n (%)**				0.184
T1	283 (22.14)	218 (22.9)	65 (19.94)	
T2	57 (4.46)	43 (4.52)	14 (4.29)	
T3	267 (20.89)	186 (19.54)	81 (24.85)	
T4	195 (15.26)	140 (14.71)	55 (16.87)	
Tx	476 (37.25)	365 (38.34)	111 (34.05)	
**AJCC.N, n (%)**				0.004
N0	324 (25.35)	261 (27.42)	63 (19.33)	
N1	631 (49.37)	453 (47.58)	178 (54.6)	
N2	103 (8.06)	69 (7.25)	34 (10.43)	
N3	64 (5.01)	44 (4.62)	20 (6.13)	
N4	156 (12.21)	125 (13.13)	31 (9.51)	
**Differentiation, n (%)**				0.084
I+II	436 (34.12)	340 (35.71)	96 (29.45)	
III+IV	632 (49.45)	464 (48.74)	168 (51.53)	
Unknown	210 (16.43)	148 (15.55)	62 (19.02)	
**Outcome**				0.168
Alive	107 (8.37)	21 (6.44)	86 (9.03)	
EAC-specific death	1009 (78.95)	269 (82.52)	740 (77.73)	
non-EAC-specific death	162 (12.68)	36 (11.04)	126 (13.24)	

Data are presented as number (percentage) for categorical data;

*Chi-square test were used for the categorical variables.

### Cumulative incidence function and Gray’s test between single-liver and bone metastasis groups

**Fig 2:**
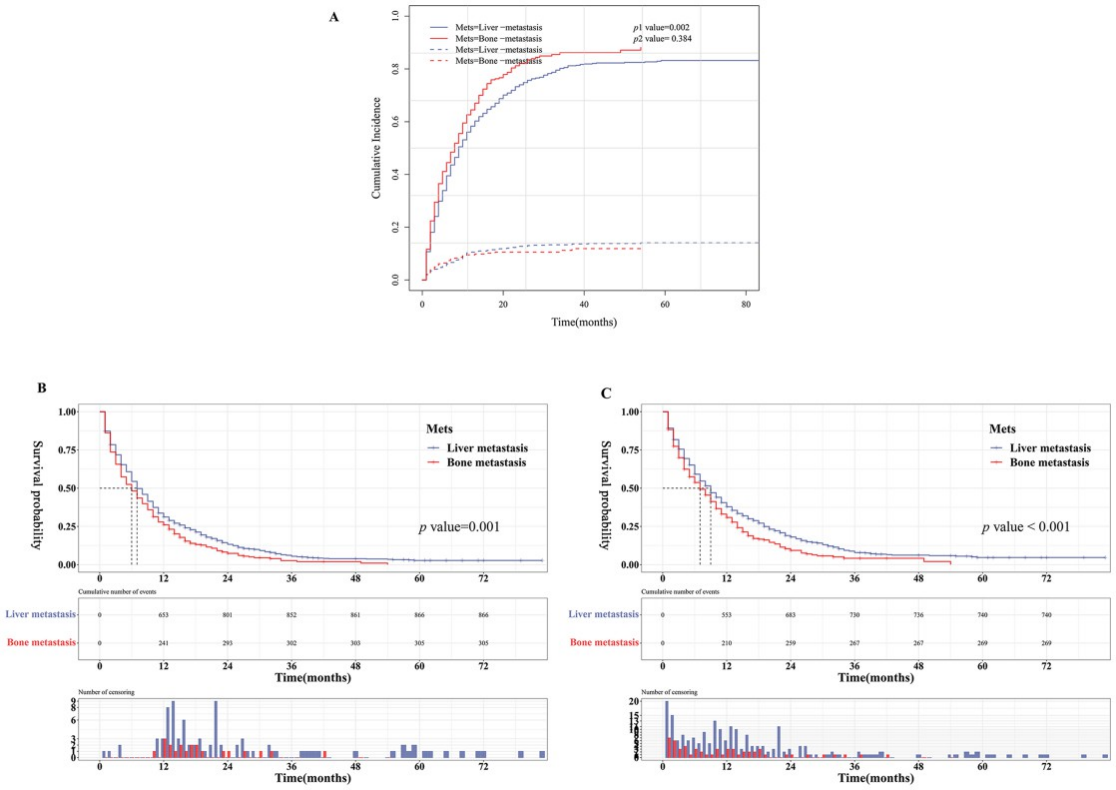
Cumulative incidence (A), OS curves (B) and CSS curves (C) for EAC patients split by metastatic sites.

The total cumulative incidence of EAC-specific death was 1009 (78.95%), and the non-EAC-specific death was 162(12.68%) (Table 1). As shown in Fig 2A, patients with single-liver metastasis had lower cumulative incidence of EAC-specific death when compared with single-bone metastasis group (Gray’s test, *p* = 0.002). Notably, the converse was true for the cumulative incidence of non-EAC-specific death although it was not shown statistically significant (Gray’s test, *p* = 0.384). The results were consistent with Kaplan–Meier analysis, patients with single-liver metastasis had better OS or CSS when compared with single-bone metastasis group (log-rank test, *p* < 0.001 for CSS and *p* = 0.001 for OS) (Fig 2B & 2C).

### Multivariate Cox regression model analysis

The independent prognostic factors in OS and CSS were evaluated using a multivariate Cox regression model. As detailed in (S1 Fig), patients with single-bone metastasis had significantly worse OS (HR,1.222, *p* = 0.004) or CSS (HR,1.265, *p* = 0.002) than with single-liver metastasis. On multivariate Cox proportional hazards regression, patients who received surgery (HR: 0.594, 95% CI: 0.375–0.939 for OS; HR: 0.562, 95% CI: 0.343–0.921 for CSS) or chemotherapy (HR: 0.345, 95% CI: 0. 301–0.396 for OS; HR: 0.366, 95% CI: 0.315–0.425 for CSS) were related to the decreased risk of death for OS and CSS. Additionally, patients who got divorced/separated (HR: 1.358, 95% CI: 1.125–1.639 for OS; HR: 1.378, 95% CI: 1.126–1.686 for CSS) or single/unmarried (HR: 1.270, 95% CI: 1.080–1.494 for OS; HR: 1.283, 95% CI: 1.078–1.528 for CSS) showed worse OS or CSS than who got married, and patients who were in Grade III-IV showed worse OS or CSS compared with who were in Grade I-II. The HRs (95%CI) were 1.357 (1.190–1.549) for OS, and 1.292 (1.122–1.489) for CSS, respectively. Moreover, male patients also showed worse OS than female patients (HR: 1.257, 95% CI: 1.042–1.516).

### Competing risk regression analysis

**Fig 3:**
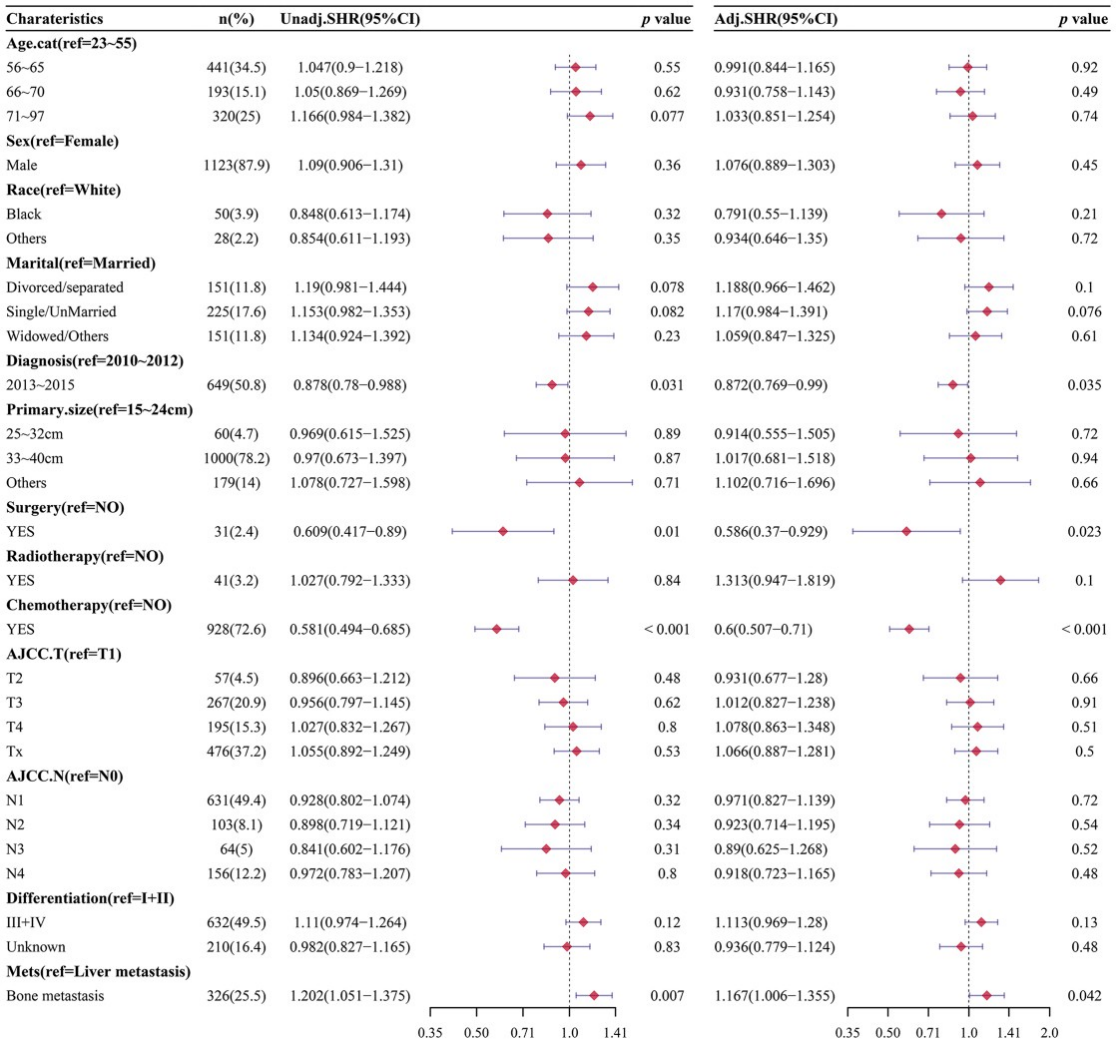
Single and multivariate Fine-Gray models for OS and CSS of EAC patients.

A competing risk regression analysis was conducted to further investigate the independent prognostic factors in EAC-specific death, (Fig 3). Patients with single-bone metastasis had worse EAC-specific death compared with single-liver metastasis (SHR: 1.202, 95%CI: 1.051–1.375) after controlling for the competing risk events, and the results were remained consistent after adjusting for other risk factors (SHR: 1.167, 95%CI: 1.006–1.355). In multivariate analysis, we also found that patients who diagnosed in 2013–2015, received surgery or chemotherapy had better EAC-specific death than those who diagnosed in 2010–2012, who did not receive surgery or chemotherapy, respectively. The SHRs (95%CI) were 0.872(0.769–0.990), 0.586 (0.370–0.929) and 0.600 (0.507–0.710), respectively.

### Subgroup analysis of the association between single-liver metastasis vs. single-bone metastasis

**Fig 4:**
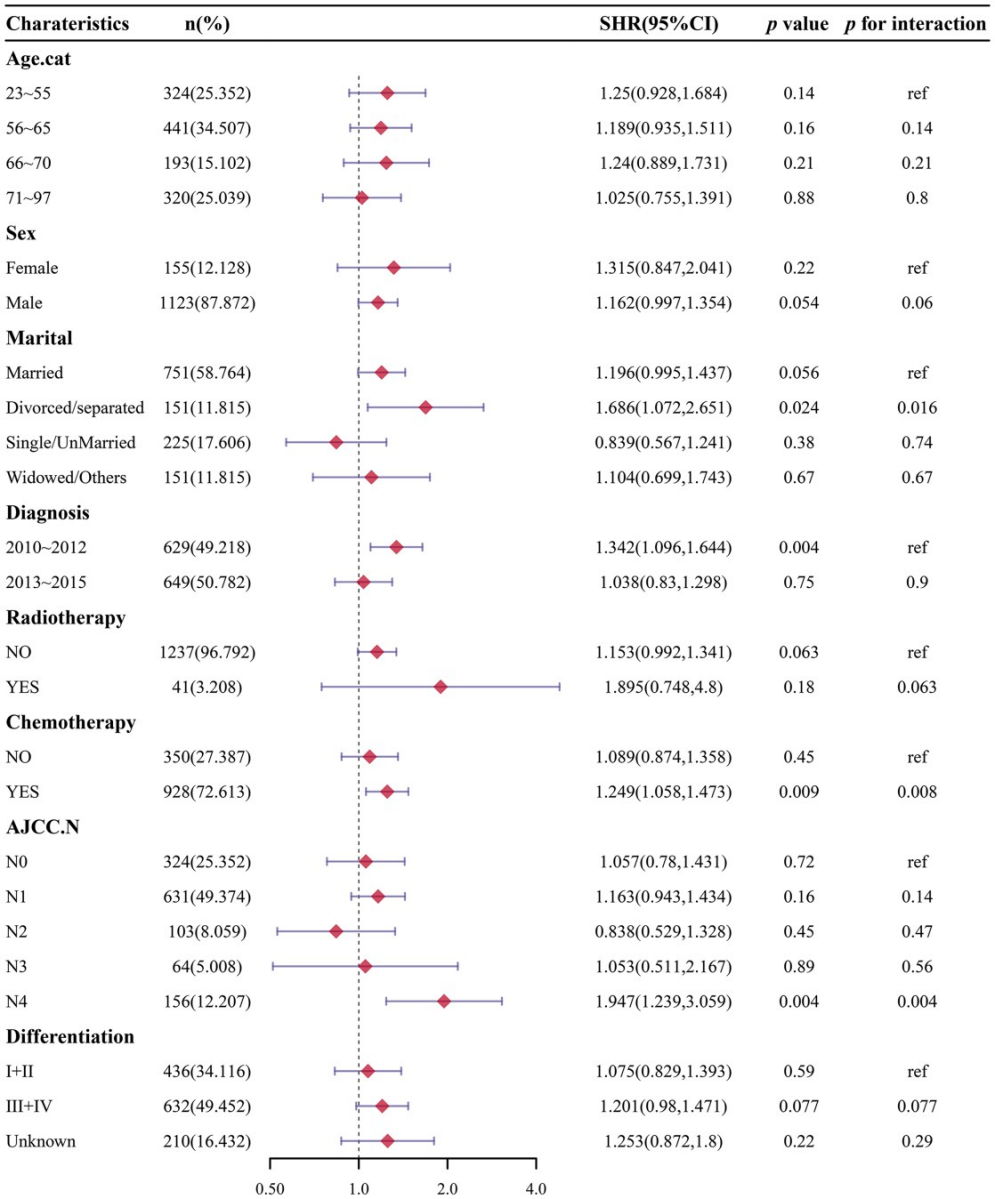
Association between single-organ metastasis by subgroups analysis (single-liver metastasis vs. single-bone metastasis).

Subgroup analysis was conducted to discriminate the most possible risk factors related with the prognosis of single-organ metastasis (single-bone metastasis vs. single-liver metastasis). Compared with patients with single-liver metastasis, factors statistically associated with higher EAC-specific death included male (SHR: 1.162, 95%CI:0.997–1.354), divorced/separated (SHR:1.686, 95%CI:1.072–2.651), diagnosed in 2010–2012 (SHR: 1.342, 95%CI:1.096–1.644), receiving chemotherapy (SHR: 1.249, 95%CI:1.058–1.473) in patients with single-bone metastasis after controlling for the competing risk events (Fig 4).

### Conditional 3‑year CSS analyses

Given 0, 1, 2 years of event-free survival, the 1-year EAC-specific death free survival probabilities were 37%, 52% and 40%, respectively for patients with single-liver metastasis (S2A Fig), and 31%, 33% and 45%, respectively for patients with single-bone metastasis (S2B Fig).

## Discussion

A number of studies have emphasized that EAC has an incidence that is rapidly rising and about 40% patients present with distant metastatic disease [19–21]. Survival of EAC patients with metastases is exceedingly poor, with less than 4% surviving 5 years [22, 23]. Single-site metastasis was the most common metastatic pattern in EAC patients, bone and liver are common organs for distant metastasis [20, 24]. However, there were limited studies on the effect of the site of DM on survival in metastatic EAC. In our study, we aimed to comprehensively analyze and compare the prognosis between BM and LM in EAC patients based on SEER database. The results indicated that bone metastasis had a significantly worse OS (median, 6 months) than liver metastasis EAC (median, 7 months). This finding in our research was consistent with previous studies that reported poorer survival outcomes for EAC patients with BM compared to those with LM or other sites of metastasis [10, 25]. However, the underlying mechanisms for this difference remain unclear.

The relationship between metastatic sites (BM&LM) and clinical characteristics, including age, sex, race, histologic grade, marital, was also discussed in our study, which would provide references for diagnosis and treatment. The multivariable Cox Regression model analysis was applied to determine the risk factors for bone metastasis development (*vs*. liver metastasis). Results showed that patients with divorced/separated, single/unmarried (*vs*. married), Grade III-IV (*vs*. Grade I-II), male (*vs*. female), surgery or chemotherapy had an increased risk to develop BM than LM. However, EAC was associated with many complications, and the study based on Cox method may overestimate the incidence of cause-specific mortality which could lead to a number of possibilities that could result in biased results. To mitigate the estimation bias and further evaluate the independent prognostic factors in EAC-specific death, a competing risk regression model analysis was conducted [26], and we found that diagnosed in 2013–2015, received surgery or chemotherapy had significantly better prognostic for patients with BM. A subgroup analysis was applied to explore the most likely risk factors associated with EAC-specific death results from the competing risk model analysis in BM compared to LM among EAC patients. Patients with BM with Divorced/separated, diagnosed in 2010–2012, receiving chemotherapy, tended to decrease the risk of EAC-specific death than LM. We further applied conditional survival methodology to estimate 0, 1, 2 years of event-free survival of LM and BM, EAC patients with BM tended to have lower relative conditional CSS.

According to previous studies, some possible mechanisms underlying the different survival outcomes between BM and LM in EAC patients were as follows. First, bone metastasis may induce more severe symptoms and complications than liver metastasis, such as bone pain, fracture, spinal cord compression, and hypercalcemia, which can impair the quality of life and performance status of patients [27]. Besides, bone metastasis may be associated with more aggressive tumor biology and higher tumor burden than liver metastasis, as evidenced by higher levels of serum lactate dehydrogenase and alkaline phosphatase in patients with BM [28]. Furthermore, bone metastasis may activate a vicious cycle between tumor cells and bone cells, which leads to increased bone resorption and/or formation, releasing various growth factors and cytokines that stimulate tumor growth and further disrupt bone homeostasis [29]. Based on these possible mechanisms, we suggest some therapeutic implications and future directions for improving the management of EAC patients with BM and LM. On the one hand, early detection and prevention of bone metastasis are crucial for reducing the morbidity and mortality of EAC patients. Therefore, more sensitive and specific biomarkers and imaging techniques are needed to identify patients at high risk of developing bone metastasis and monitor their response to treatment [30]. On the other hand, effective treatment of bone metastasis should aim to relieve symptoms, prevent skeletal-related events, inhibit tumor growth, and prolong survival. Therefore, a multidisciplinary approach that combines systemic therapy (such as chemotherapy, targeted therapy, immunotherapy) with local therapy (such as surgery, radiotherapy, ablation) and supportive care (such as bisphosphonates, denosumab, analgesics) is recommended [10].

Our study pioneers the application of competing risk analysis in the context of mEAC. By considering both BM and LM as competing events, we move beyond traditional survival analyses. This approach allows us to explore how different metastatic sites impact patient outcomes simultaneously. The integration of competing risk methodology provides a more nuanced understanding of mEAC prognosis. However, there were several drawbacks such as its retrospective nature and possible patient selection bias, which is common in the retrospective design of the study. In addition, this study is based on a US cancer registry. Therefore, specific observations related to the influence of other regions are not available. Previous studies have indicated a clear influence of socioeconomic factors on the outcomes of patients with EAC [31].

## Conclusions

In this study, we harnessed the multivariate Cox regression, Kaplan-Meier analysis and competing risk regression models to explore the prognosis of mEAC. Our study indicated that patients with BM experience a deteriorated prognosis compared to those with LM. Furthermore, we identify surgery and chemotherapy as protective factors for mEAC patients. Our findings may help distinguish high-risk patients with BM and LM and evaluate prognosis in mEAC patients. These results indicate the need to review our current research strategies for mEAC patients and such discussions should be a part of the shared decision-making process. We also discussed the possible mechanisms and therapeutic implications of the different survival outcomes between BM and LM in EAC patients, and suggested future directions for improving the management of EAC patients with bone metastasis. For instance, mEAC patients with BM may require more aggressive management strategies, while those with LM might benefit from targeted therapies. This personalized approach enhances the precision of treatment decisions. We hope that our study could provide insights and references for clinicians and researchers who are interested in this field.

## Supporting information

S1 Fig Multivariate Cox proportional risk model for OS and CSS of EAC patients with single-organ metastases. (DOCX)

S2 Fig Conditional survival analysis for patients of single-liver metastases (A) and single-bone metastases (B) for CSS. (DOCX)

S1 Data Study data. (XLSX)
