# Supplementary material for: Comparing the prognosis of esophageal adenocarcinoma with bone and liver metastases: A competing risk analysis
Source: PLoS One. 2024 Sep 25;19(9):e0303842. doi: 10.1371/journal.pone.0303842 (PMC11423978; doi:10.1371/journal.pone.0303842)
Supplement: S1 Fig — (DOCX) [file pone.0303842.s001.docx]

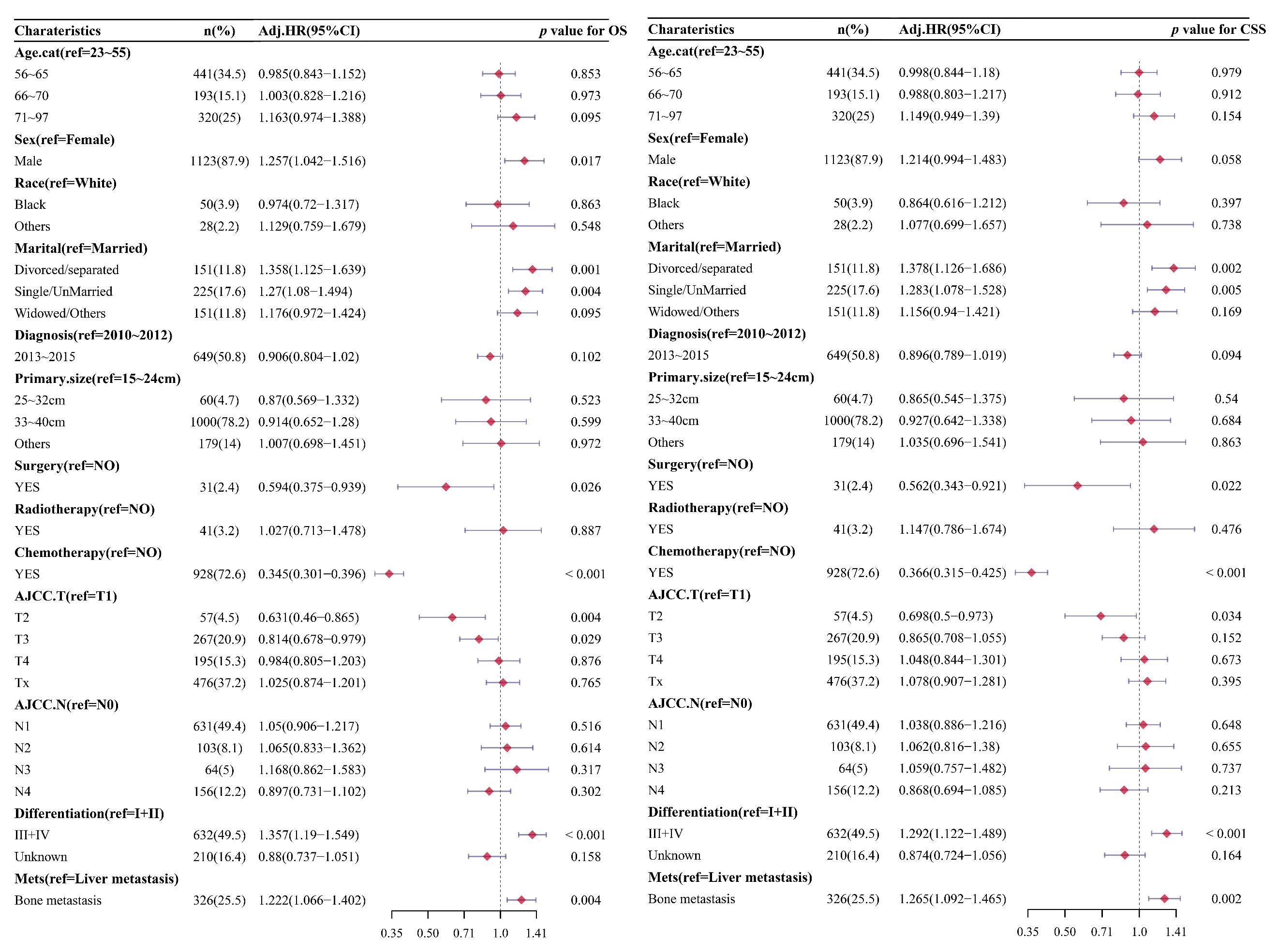


**Fig S1.** Multivariate Cox proportional risk model for OS and CSS of EAC patients with single-organ metastases.
