# Supplementary material for: Comparing the prognosis of esophageal adenocarcinoma with bone and liver metastases: A competing risk analysis
Source: PLoS One. 2024 Sep 25;19(9):e0303842. doi: 10.1371/journal.pone.0303842 (PMC11423978; doi:10.1371/journal.pone.0303842)
Supplement: S2 Fig — (DOCX) [file pone.0303842.s002.docx]

**Fig S2. Conditional survival analysis for patients of single-liver metastases (A) and single-bone metastases (B) for CSS.**
